# Pathways between foodways and wellbeing for First Nations Australians

**DOI:** 10.1186/s12889-024-18005-y

**Published:** 2024-02-16

**Authors:** Kate Anderson, Elaina Elder-Robinson, Megan Ferguson, Bronwyn Fredericks, Simone Sherriff, Michelle Dickson, Kirsten Howard, Gail Garvey

**Affiliations:** 1 School of Public Health, Faculty of Medicine, The University of Queensland, Herston, QLD Australia; 2 Office of the Deputy Vice Chancellor (Indigenous Engagement), The University of Queensland, St. Lucia, QLD Australia; 3 The Poche Centre for Indigenous Health, Faculty of Health and Biomedical Sciences, The University of Queensland, St. Lucia, QLD Australia; 4 School of Public Health, Faculty of Medicine and Health, The University of Sydney, Sydney, NSW Australia; 5 The Sax Institute, Sydney, NSW Australia; 6 The Poche Centre for Indigenous Health, Faculty of Medicine and Health, The University of Sydney, Sydney, NSW Australia; 7 Menzies Centre for Health Policy and Economics, Faculty of Medicine and Health, The University of Sydney, Sydney, NSW Australia

**Keywords:** Aboriginal and Torres Strait Islander people, Indigenous, Wellbeing, Nutrition, Food, Foodways, First Nations, Australia

## Abstract

**Background:**

Supporting the health and wellbeing of Aboriginal and Torres Strait Islander peoples (hereafter respectfully referred to as First Nations peoples) is a national priority for Australia. Despite immense losses of land, language, and governance caused by the continuing impact of colonisation, First Nations peoples have maintained strong connections with traditional food culture, while also creating new beliefs, preferences, and traditions around food, which together are termed *foodways*. While foodways are known to support holistic health and wellbeing for First Nations peoples, the pathways via which this occurs have received limited attention.

**Methods:**

Secondary data analysis was conducted on two national qualitative datasets exploring wellbeing, which together included the views of 531 First Nations peoples (aged 12–92). Thematic analysis, guided by an Indigenist research methodology, was conducted to identify the pathways through which foodways impact on and support wellbeing for First Nations peoples.

**Results and conclusions:**

Five pathways through which wellbeing is supported via foodways for First Nations peoples were identified as: *connecting with others through food; accessing traditional foods; experiencing joy in making and sharing food; sharing information about food and nutrition;* and *strategies for improving food security*. These findings offer constructive, nationally relevant evidence to guide and inform health and nutrition programs and services to harness the strengths and preferences of First Nations peoples to support the health and wellbeing of First Nations peoples more effectively.

## Background

Aboriginal and Torres Strait Islander peoples (hereafter respectfully referred to as First Nations peoples), are the first peoples of Australia and comprise over 300 distinct and diverse groups, each with their own culture, language, beliefs, and customs [1]. It is estimated that First Nations peoples have lived and thrived in Australia for over 60,000 years and have continued to maintain deep and complex knowledge systems to support their health and wellbeing [1]. The comparatively recent impacts of European colonisation over the past 250 years have produced substantial losses of land, language and governance for First Nations peoples and have resulted in significant inequities for First Nations peoples across a range of health and wellbeing metrics [2]. Despite the immense losses, First Nations knowledge systems have survived and continue to support the health and wellbeing of Australia’s First Nations people [1]. Redressing the inequities facing First Nations peoples is an urgent national priority; one that requires guidance and direction from First Nations peoples to find effective solutions and avoid further injury [3, 4]. Ensuring that the health and wellbeing of First Nations peoples and their traditional knowledge systems are supported to thrive is a human rights imperative, [5] and offers opportunities for all Australians to learn and benefit from these traditional knowledge systems and cultures.

The experience and conception of wellbeing is culturally bound, [5] therefore accurate understandings of wellbeing from First Nations peoples’ perspectives is a critical foundation for any program or policy aiming to improve health and wellbeing for First Nations peoples. The wellbeing for First Nations peoples is an interwoven and multifaceted construct, with the individual’s wellbeing closely tied to the wellbeing of one’s family, community, and Country (land/territory), [6, 7] and is distinct from common biomedical and Western conceptions of wellbeing that focus on the individual [8]. One key component within this interwoven construct of wellbeing is the attainment of *holistic health*: a multidimensional experience of wellness that includes connection to culture, land, family and community [9]. The opportunity to have good health is a fundamental element of health equity and human rights discourse, [5] and understanding how to support holistic health is an integral component in improving wellbeing for First Nations peoples. Central to the concept of holistic health is food and foodways [6, 9]. Foodways are the cultural, social, and economic practices of people and communities, which affect the consumption, procurement, choice, and preferences of food [10]. Foodways hold important symbolic, cultural, and emotional value in the lives of First Nations peoples [6, 11]. The importance of traditional diets is highlighted in the transition from traditional to contemporary diets which has seen reduced opportunities for physical activity and sharing of cultural knowledges associated with worsened health outcomes [6, 12]. Recent qualitative studies with First Nations adults’ found that wellbeing was greatly improved when physical activity and food procurement intersect with traditional and culturally-oriented activities [7].

The connection between foodways and wellbeing must be considered in the context of the dynamic relationship between First Nations peoples and Country, the ongoing impacts of colonisation, and the rapidly changing socio-political environment in which First Nations people live. The sophistication of traditional First Nations foodways are evident in agriculture practices, the knowledge of which was passed down through thousands of generations [13]. The complexity of this relationship to Country and food systems has been repudiated and misrepresented in “hunter-gatherer” colonial narratives. First Nations truth-telling is beginning to reshape and rectify this narrative, [13] with increased awareness around the intentional agricultures that have been part of First Nations practices for thousands of years. This new narrative aides comprehension of the extent of colonial destruction of First Nations foodways, including the damage to many traditional food systems; environmental degradation due to introduced plants, animals, agricultural practices, and mining; changes in nutritional composition of available foods; the use of food and water as a weapon of genocide; and the use of food as a form of colonial control, which have all been devastating elements of colonial influence on First Nations peoples [14]. The culmination of these historical dietary factors for many First Nations peoples today, combined with socioeconomic, environmental and geographic factors, is a contemporary diet high in refined carbohydrates, and processed foods, which facilitate longer shelf-life and ease of transport [14–16]. Such foods are quite disparate from those of pre-colonisation diets which were rich in plant foods and lean meats [14, 17, 18]. Barriers to accessing nutritious food and additional food security issues are increasingly affecting many First Nations communities peoples, impacting on the health and wellbeing of First Nations peoples [12, 14, 19–21].

Within this historical and cultural context, there is a pressing need to better understand and leverage how foodways can, and do, support wellbeing for First Nations peoples. Much of the recent research with First Nations communities has been focused on the efficacy of nutrition interventions, [22–24] and addressing food security issues, including in daily life for growth, development and improving health status, and in times of natural disasters and crisis, for example during the COVID pandemic [25, 26]. There is an increasing need to understand of how the intersection of foodways and wellbeing can offer a powerful, constructive, and strengths-based guide to inform programs and services to support the health and wellbeing of First Nations peoples more effectively.

The current study conducted secondary qualitative data analysis of a combined dataset from two national studies to understand First Nations peoples’ perspectives on the ways in which foodways and wellbeing are connected and the pathways via which foodways are understood by First Nations peoples as supporting and promoting their holistic health and wellbeing.

## Methods

This paper presents a secondary analysis of qualitative data collected during Phase 1 of the What Matters 2Adults (WM2Adults) research project [7, 27] and Phase 1 of the What Matters 2Youth (WM2Youth) research project. Both projects aimed to develop nationally relevant measures to assess wellbeing for First Nations adults and youth (aged 12–17 years), respectively. This secondary analysis aimed to explore First Nations peoples’ perspectives on interconnections of foodways and wellbeing, in order to identify and describe the pathways through which foodways support wellbeing for First Nations peoples.

### Indigenist methodology

Our team intentionally works to honour and prioritise First Nations peoples ways of knowing, being and doing in all aspects of our work [28]. This research used a strengths based, Indigenist approach to ensure the prioritisation and elevation of the voices and values of First Nations peoples, underpinned by Indigenist research methodologies [29–31]. There is focus on the autonomy and leadership of First Nations peoples while understanding the cultural, social, and political realty of First Nations lives across the entirety of the research [31]. For example, First Nations peoples were included in all aspects of the project, a highly experienced First Nations health researcher (GG) provided research leadership, and First Nations Advisory Groups were established for each of the projects to ensure the provision of continual feedback and advice to the research team.

### First nations governance

First Nations Project governance and Advisory Groups were established at the inception of both the WM2Adults and WM2Youth projects and each of these groups consisted of representatives of key First Nations stakeholder groups and community members. These groups have guided the overall program of work, from inception to dissemination and detail about these are reported in the study protocols [27, 32].

### Positionality

Our team is comprised of First Nations and non-First Nations researchers. As a team, we work consciously and collaboratively to conduct all aspects of our research in ways that prioritise and support the culture, strengths, and wellbeing of First Nations peoples. This principle underpins our research processes and guides how First Nations peoples are reflected in the research outputs and literature that originate from our work. Our team acknowledges the importance of reflexively considering and describing our backgrounds, perspectives, and values that we each bring to the project [33, 34]. The research team includes First Nations researchers (GG, MD, SS, BF), qualitative researchers (GG, MD, KA, SS, MF, BF), a junior researcher and health practitioner (EE) and a health economist (KH) working in First Nations Health Research.

### Participants and data collection

**Fig. 1:**
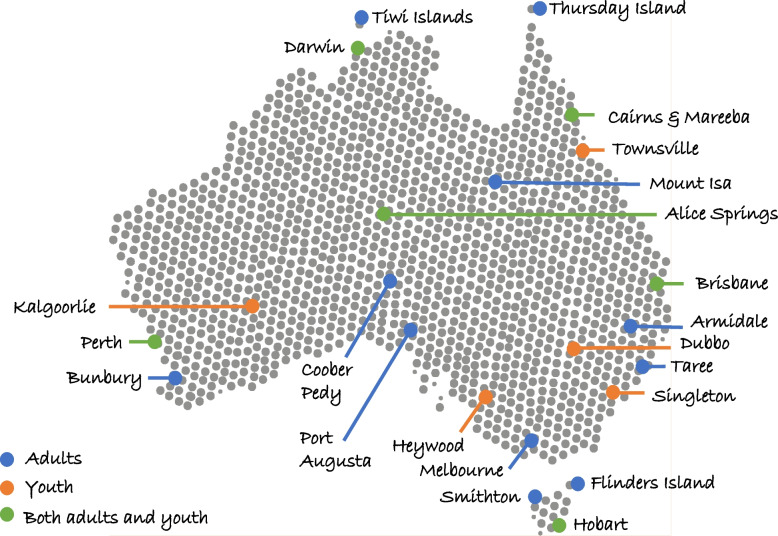
Yarning Circle sites

Detailed methods for WM2Adults are reported elsewhere [7, 27]. In brief, recruitment of First Nations adults (18 years or older at time of recruitment) was undertaken through purposive recruitment strategies, across Australia, between September 2017 to September 2018. Participants were recruited from a range of locations across Australia (see Fig. 1) and invited to take part in Yarning circles at identified sites, led by First Nations researchers [28, 35–38]. Yarning is a culturally-recognised and appropriate form of communication, and in this context is as defined by Bessarab and Ng’andu (2010) [35] as a type of conversation, which can be applied to health and well-being research environments. In the adult Yarns, participants were asked to describe and explore the parts of life that impacted on wellbeing, guided by a First Nations facilitator. Yarns were audio-recorded and conducted face to face with ~ 8–10 Aboriginal and Torres Strait Islander adults, taking between 60 and 120 min. The facilitator utilised a Yarning guide to understand what research to listen for, and expand on, during the Yarn [38].

A detailed description of methods for WM2Youth are reported elsewhere [32]. In brief, First Nations youth (aged 12–17 years) were invited to participate in PhotoYarning sessions, recruited between October 2021 and September 2022 from a range of locations across Australia (see Fig. 1). Recruitment was undertaken with the assistance of partner organisations who run community-based youth programs, with partner organisations responsible for facilitating recruitment, gaining consent, and organising Yarn venues. The PhotoYarning method was used with young participants due to its visually engaging and culturally relevant elements [39]. After an initial Yarn to inform participants of the PhotoYarning method, participants were given digital point-and-shoot camera to take photos of things in their life that relate to wellbeing. In a follow-up Yarn, participants were asked to share and reflect on photos they had taken, and to describe and explore the parts of life that impacted on wellbeing. Yarns were audio-recorded, facilitated by either two First Nations facilitators or one First Nations and a non-Indigenous facilitator. A modified Yarning guide was used by facilitators to ensure participants had opportunity to comment on parts of life that impact wellbeing if these were not covered in photographs.

Transcripts from all Yarns conducted as part of the WM2Adults and WM2Youth projects were imported into NVivo12 software for analysis [40].

### Ethics

Ethics approvals for secondary analysis for WM2Adults and WM2Youth were obtained from relevant Ethics Committees, including Human Research Ethics Committee of the Northern Territory Department of Health and Menzies School of Health Research (WM2Youth: 2020–3850) and University of Sydney Human Research Ethics Committee (WM2Adults: 2017/724). Approvals for the original studies are reported in the respective study protocols [7, 27, 32].

### Data analyses

Secondary data analysis was conducted on the WM2Adults and WM2Youth Phase 1 qualitative data sets. Researchers conducting the analysis were involved in the two parent studies. Two researchers (EE, KA) reviewed the WM2Adults and WM2Youth transcripts using a inductive and reflexive thematic analysis approach, [41] grouping quotes which spoke to similar ideas and synthesising draft thematic headings accordingly to describe the interrelationship between foodways and wellbeing for First Nations adults and youth. Emerging thematic headings were shared and discussed initially with First Nations researchers (GG, MD) and then refined in consultation with other researchers (KH, MF, SS, BF) via email discussions, due to the wide geographic spread of the research group. There were three revisions rounds with co-authors prior to confirming the five pathways between foodways and wellbeing explored in the results below. While the analysis process did not intend to explicitly and solely compare differences between age groups, iterative comparisons between adult and youth participants were made throughout the coding and thematic analysis process where they arose.

## Results

### Participant characteristics

Data from 531 First Nations peoples in Australia was included in this analysis: 359 adults (18–92 years) and 172 youth (12–17 years). Ages ranged between 12 and 92 years, with a median age of 36 years. The sample was mostly female (61%), identifying as Aboriginal (85%), and spoke predominantly English language at home (87%). The largest proportion of participants lived in Queensland (22%), followed by Western Australia (21%) and the Northern Territory (18%). More detailed demographic information about participants is presented in Table 1. The data was collected from 71 (53 WM2Adults and 18 WM2Youth) Yarning Circles across 39 sites (25 WM2Adults and 14 WM2Youth sites) in seven Australian states and territories between September 2017 and September 2022. More details of sites are presented in Fig. 1.

**Table 1:** Demographic participant data

**Demographics**		
Age range (years)	12–92	Median—36
Age	N	%
12–19 years^a^	183	35.40
20–29 years	45	8.70
30–39 years	51	9.86
40–49 years	63	12.19
50–59 years	73	14.12
60–69 years	68	13.15
70–79 years	27	5.22
≥ 80 years	7	1.35
Gender		
Male	203	38.23
Female	326	61.39
Other	2	0.61
Main language		
English	452	87.26
Other	66	12.67
Indigenous status		
Aboriginal	335	85.03
Aboriginal and Torres Strait Islander	37	9.39
Torres Strait Islander	22	5.58
State		
New South Wales	95	18.03
Western Australia	113	21.44
Queensland	116	22.01
Northern Territory	97	18.41
Victoria	33	6.26
Tasmania	43	8.16
South Australia	30	5.69
Remoteness^b^		
Major city	78	15.35
Inner Regional	139	27.36
Outer Regional	179	35.24
Remote	62	12.20
Very Remote	50	9.84

^a^This proportion is large as it contains the entire participant sample from the WM2Youth study

^b^Remoteness was classified using postcode and locality data within the Accessibility/Remoteness Index of Australia (ARIA) 2016 [42]

### Qualitative findings

While food is essential for the survival and health of all people, the stories and descriptions shared by participants revealed a series of deeply valued and culturally embedded pathways by which foodways support and enrich the wellbeing of First Nations peoples. Our thematic analysis identified the following five pathways through which foodways promote the wellbeing of First Nations adults and youth: *(1) connecting with others through food; (2) accessing traditional foods; (3) experiencing joy in making and sharing food; (4) sharing information about food and nutrition; and (5) strategies for improving food security*. Theme two focuses on traditional foodways, whilst theme four focuses on navigating contemporary foodways. Themes one, three and five include elements from each. Each of these five pathways are described in detail below, with the inclusion of direct quotes from participants to exemplify and illuminate the nuances within each.

As it is not the aim of this paper to specifically compare or contrast the views of adult and youth participants, our thematic analysis largely combines the views across these age groups. However, where the views of adult or youth groups focused on a particular topic/s that did not strongly emerge for the other age group, this is described below. Similarly, direct quotes are labelled with the region where the person participated in a Yarn, and whether the participant was an adult or youth.

### Connecting with others through food

Participants shared many stories and descriptions about the ways in which food provided highly valued opportunities to spend time and connect with family, friends, and other people in their communities. The communal sourcing, sharing, preparing, and eating of foods were commonly reported by participants. Participation in these types of activities with others was described as important for deepening social and cultural connections, which in turn was identified as a key element in supporting and strengthening both individual and collective wellbeing.

Participation in communal activities like hunting, growing, and collecting traditional foods and ingredients were activities that were highly valued by both adult and youth participants. Spending time on Country with friends and family while engaging in these types of activities afforded opportunities to spend time on Country enjoying each other’s company, developing and honing hunting, foraging, and cultivating skills, and sharing and maintaining cultural traditions. Engaging in food-related activities was deeply enmeshed with fostering an individual’s and a communal sense of connection and identity, as well as instilling a deep connection with, and appreciation of, nature and Country.

*Just fish, catch crab, worm and oysters, pipis, I’d go fishing, yeah…. Got a good feed and a lot of my friends there. You go down the beach and go prawning; we used to come home with a big bucket full. (Adults, Taree)*

*It’s amazing how* [the community garden] *is now, and how it was only started off with a few foods…. There’s people from all over the community that work there …. I like going there. I come down there for food. (Youth, Kuranda)*

Participating in such activities was especially valued by young people who attended boarding school and were often far away from their home and families. For these young people, going out hunting and collecting food with friends and family was relatively rare and was greatly missed.

*Going out shooting with my older brothers, catching bush tucker, and, yeah….because I’m not always with them, it feels good to be back with them. (Youth, Heywood)*

The importance of engaging in activities like hunting and foraging to ensure cultural continuity and maintain familial and community connection was also shared by participants. Some adult participants lamented the fewer opportunities there were for their children and grandchildren to join into this type of cultural communal activity, which were dwindling due to circumstances such as, younger family members moving away from communities, children being at boarding schools, and the loss of access to land.

*We speak to all our grandchildren on the phone, that’s not the same as being with them. And especially with mutton bird, like, we’re losing that and it’s sad, we have not got the young people to come mutton birding because plus the cost of coming over and I think some of them are going to miss out because you need to start your children at a very young age, mutton birding. (Adults, Flinders Island)*

Many participants described their everyday connections with family and friends over food as key events that supported their wellbeing. Sometimes these occurred at home with family and other times this was in the context of during and after school hanging out with friends.

*This morning we had pancakes and most Sundays we have pancakes, and all of us just sit there, have our pancakes, maybe watch a movie, and start our day with pancakes. (Youth, Singleton)*

In reflecting on their wellbeing, participants spoke about certain foods, meals, and celebrations as holding special memories and meanings for them. Such foods and meals were described as opportunities to spend relaxing and enjoying time with loved ones, as well as remember loved ones who had passed away.

*…me and my nan would always eat strawberries dipped in chocolate together. She would always bring a bucket of chocolate and strawberries, no matter where we were. (Youth, Alice Springs)*

*We have our own families, but we always know, …nothing else matters when everyone is together over Christmas dinner. (Adults, Darwin)*

*Food has been a big part of* [our] *family because mum likes to cook for us a lot, especially for special occasions like birthdays and Christmases. So, things like that bring us together with family. (Youth, Newcastle)*

For those participants who were unable to connect with others for special meals and celebrations, they conveyed that this caused great sadness and strongly affected their wellbeing. Some adult participants, who were unable to live in their home community due to health concerns, such as the need to access renal dialysis, described the sorrow they felt at not being able to join family for meals.

*If we need transport to go out just for a day or funeral service, Sorry Business, it’s hard. It’s hard on Christmas night or Christmas Eve, even for Sorry Business too. Yeah, all the things that go in that nice meal. (Adults, Alice Springs)*

### Accessing and consuming traditional foods

The ability to source and eat traditional foods, known in many communities as *bush tucker*, was described by participants as an important pathway to fostering wellbeing. Eating fresh and locally sourced foods, particularly those that are part of a traditional diet, was described by participants as offering powerful benefits to peoples’ health and wellbeing.

*I find having traditional foods improves your health and the way you live. It sort of makes you feel more healthy. (Adults, Melbourne)*

Traditional foods were regarded as beneficial; however, participants highlighted that diets and access differ vastly across cultural groups and regions. Many individuals felt strongly drawn to eat foods that are connected to their own Country and culture, as some considered these to be the most nourishing and health-giving for them. Foods have direct and indirect connections to people linked through place, Country [43, 44]. As such foods are a direct connection to one’s Country and a further extension the self within Country, and how everything is connected [45]. Bob Morgan explains it this way, “… my culture and worldview is centred in Gumilaroi land and its people, it is who *I am* and will always be. *I am* *my country*.” (p. 204) [46]. Eating food from one’s Country is an affirmation and reiteration of the self, and a continuation of the lifeforce of self and Country, with emphasis of connectedness across all aspects of life. The quotes below highlight this lived reality.

*Like I am Barkly* [inland region in the Northern Territory], *this lady is saltwater. And we get different food, different tucker. (Adults, Darwin)*

*We’re sea water people. We need fish three or four times a week. Oysters. Crab. Turtle. Dugong. And that’s where our husband is, he is out now. So, we will get fish tomorrow. (Adults, Darwin)*

The ability to source one’s own food directly from Country was desirable for many participants, due to the identified nutritional qualities of traditional foods.

*You know, wallaby and stuff and the bush foods and all of seafood …. that is the key to our physical health. To those chronic diseases that we suffer because of sugar and jam and flour, pastry. (Adults, Hobart)*

*‘Bush tucker. I reckon bush tucker…. kangaroo meat is good for Aboriginal people, because it’s low in fat.’ (Adults, Port Augusta)*

Avoiding refined foods like sugar and flour, and instead eating traditional foods, were described by adult participants as the key to maintaining health and wellbeing and avoiding chronic diseases.

Youth participants also shared their views about their preference for and the importance of eating traditional foods. While youth participants did also share stories of hanging out at fast food establishments and baking cakes and biscuits, they also described eating foods that were caught or grown by themselves, or by family and friends, as being particularly valued and appreciated.

*Q: Just proud of your culture, that’s really important. And what about food, you were talking about food?*

*What kinds of foods do you get to eat?*

*P: Oh, underground food. I’d say turtle. You cook it underground. (Youth, Townsville)*

Engaging in the hunting and collecting of traditional foods was also described as important to wellbeing as they offered opportunities for building and honing culturally grounded skills associated with hunting, survival, and food preparation.

*Q: And so you go hunting because you like it.*

*P3: Yeah.*

*Q: And it also gives you food?*

*P3: Yeah.*

*Q: Do other people go hunting for tucker? Yeah.*

*P1: Yeah. I go hunting.*

*Q: What sort of things do you hunt?*

*P1: Turkey, kangaroo, sometimes bullock, I think.*

*Q: And so what is about hunting? Does it connect you to culture as well, or is it something different? Is it more about food?*

*P1: Yeah, food mainly.*

*P3: And you learn things from it as well. Like learning how to skin a kangaroo and that.*

*Q: Oh, so you learn some skills.*

*P1: Learn how to make spear. (Youth, Karratha)*

For some, being able to source traditional foods was dependent on the accessibility of the lands and waters where these foods are found. Government restrictions and the need for permits for some areas were described as barriers that stifled access to traditional hunting and food collecting sites. Moreover, prohibitive travel costs made getting to many desirable sites and locations unfeasible. The detrimental effects of these barriers on the wellbeing of First Nations people were described as deeply frustrating, and the cultural loss associated with the inability to access these foods and participate in the activities around these foods weighed heavily on the minds of many.

*It goes back to that identity, it’s about our rights to access that stuff* [traditional foods] *on our country. What might be locked up and owned by Parks and Wildlife, but some people in our community and this is about giving our young people, and some of our old people, about working on our confidence in who we are and our rights to access all this stuff. … Well, if I want a feed of mutton birds, I’m going to have a feed of mutton birds …This is my right. (Adults, Hobart)*

Even when physical access to hunting and collecting food was possible, participants spoke about the environmental barriers such as habitat degradation and loss, and the proliferation of feral animals causing reduction and extinction of native animals. These environmental impacts on food and food-related activities were keenly felt as substantial obstacles to First Nations peoples being able to source foods that were valued for their cultural associations and nutritional benefits.

*We used to provide kangaroo meat. And we used to go up on the hills here and we used to go shoot the kangaroos. But now we can’t go up there unless you have approval. (Adults, Perth)*

*The country before the white fellows come here, even the white fellows was here, they bought [sic] in the fox, they bought [sic] in the cats, this bit here had a lots of lizards from a sleeping lizard, and now you can’t find even little ones like that. Now you can’t find one. White fellow took fox, cats out there, most of them cats, they about this big, that tall, like lions and tigers they’re walking round. They’re killing the little pigs what we ate. (Adults, Port Augusta)*

For adult participants, a sense of grief was sometimes expressed about the loss of types of foods and foodways from their childhood and youth. Commonly, these lost foods and activities were associated with the sourcing of food directly from the environment on and within Country. The prevailing contemporary reliance on pre-prepared food from shops and supermarkets was described as detrimental to health and food security.

*Like, looking back from when my Dad did gardening, we eat food from the ground, not from the shops. He goes fishing, we eat fish. The only thing we buy from the shop would be a bag of rice and a tin of flour – bag of flour. And everything else comes out of the ground. We used to be gardeners. We grew up healthy. We had everything. We weren’t hungry. (Adults, Port Augusta)*

### Experiencing joy in making and sharing food

While participants explained that access to traditional foods and practices was highly valued, there were also stories shared about preparing all types of food and getting together for meals as being greatly valued in terms of time spent having fun, laughing, and joking with family. The experience of sharing a meal with family and friends offered joy to participants and strengthened their sense of wellbeing. These experiences of preparing and sharing favourite and/or familiar foods with people who are important in their lives were described as having positive influences on people’s lives and as conveying immense benefits to peoples’ wellbeing. The connective quality of food and food practices can be seen to repair some of the wellbeing impacts owing to the loss of traditional foodways, enabling First Nations peoples to continue to connect in new ways with family and friends through food.

*We are Aboriginal. We all feel that same belonging. It is just that connection and I feel at home . . . I just went to one of the family birthdays and I felt the family connection there. Just going and having a feed together. A yarn and a laugh, singing and laughter is our thing. Humour. (Adults, Tamworth)*

Youth participants spoke about their experiences of joy when cooking and eating foods that they liked. They described cooking as a relaxing and creative activity, and they garnered a sense of accomplishment from making delicious food. For youth participants, being able to help and contribute to the cooking of family meals provided them with a sense of responsibility and agency.

*Because it’s basically like, my happy place, and whenever like – food’s basically like, what I love mostly, because it’s – like, it’s going to be there whenever you want it. (Youth, Alice Springs)*

*I like making food. I like cooking the best. (Youth, Darwin)*

*Q: Why do you think cooking’s good for your wellbeing.*

*P: It’s a bit like imagination, like how you want your food to be. (Youth, Darwin)*

*Me and my mum do meal prep on the weekends for the school. I like it, it’s good. . . I try and cut out all bad carbs and stuff. . . Eat healthy. (Youth, Mareeba)*

*Cooking a big feed. Because I get to eat it afterwards. It like* [cooking], *I don’t know, just makes me happy. (Youth, Mareeba)*

Traditional cooking, for example via an open fire or earth oven, or curing, along with eating spaces were also described by some youth as centrally important in supporting their wellbeing. The process of cooking within an earth oven or underground can take the best part of day in terms of preparing the oven, gathering the materials needed, and ensuring the temperature is appropriate to cook food evenly and safely. Youths’ enjoyment of spending time connecting with family and community in the context of engaging in and learning about their culture was particularly valued.

*P: That’s the Kup-murri place* [Torres Strait Islander term for a traditional underground/earth oven]*. It’s when you make food with lots of them, yeah.*

*Q: So why is this important?*

*P: Because it’s a part of my culture.*

*Q: It’s a part of your culture. What do you cook there?*

*P: Damper. We sometimes cook potatoes or put pumpkin in there. And some meat.*

*Q: And how does that make you feel when you are all around, cooking and waiting for the food?*

*P: Excited.*

*Q: Eat some good tucker?*

*P: Yeah.*

*(Youth, Kuranda)*

### Sharing information about food and nutrition

First Nations’ peoples are navigating food environments with contemporary, introduced foods, often with reduced access to traditional foods as described above. Adult participants spoke about the importance of having access to current information and guidance about nutrition and diet, especially for those trying to prevent or manage chronic disease. This nutrition information was in reference to contemporary foodways. It was flagged that while many people try hard to eat well and stay healthy, they do not always have access to the best information to support healthy food preparation and eating habits.

*People think that if they’re having vegies that’s right, but it’s how you cook it. … how are you cooking your meat. … Your intake of how much. So really, it’s the education of that for our mob doesn’t get talked enough. Balance it out. (Adults, Tamworth)*

Navigating complex and varying information and advice around nutrition and healthy foods was described as a struggle for some people. The uncertainty and misunderstanding around nutrition information was regarded by some adults as a substantial barrier to achieving health and wellbeing.

*Well, they’re always changing the rules so if you’re not up to date with all the changes in food or you know, it’s all to do with health and yeah, the more understanding we have like the better health outcomes we’ll have. (Adults, Darwin)*

Being a good role model for children around eating healthy was an important factor in their own [adults] wellbeing and identity, as well as for supporting the health and wellbeing of younger generations.

*I look at my son, I see the effects of me thinking healthy and being that role model…. it’s good for the generations and people are more happy when they’re healthy. (Adults, Melbourne)*

Having access to information about nutrition was also identified as a benefit to wellbeing by youth participants, who described their appreciation of the fresh fruit and healthy foods provided by First Nations school programs. These young participants reported feeling encouraged and supported by the mentors from these programs to think about their diet and make healthy food choices.

*That’s good because* [program mentors] *has fruit here as well so they encourage you to eat healthy. (Youth, Darwin)*

### Strategies for improving food security

The need to ensure that families and communities have adequate food to eat was spoken about by adults and some youth participants as a critical component in maintaining wellbeing. While there were some commonalities in the strategies used to ensure food security across the Yarning sites, there were notable differences in how food insecurity was experienced by participants and how the experience impacted the wellbeing of participants living in regional and remote locations, compared with those living in urban settings.

The cost and availability of healthy foods was described by participants as a significant factor that affects food choice and the ability to eat nutritious and desired foods. Having enough money to buy good quality, nutritious food to feed yourself and your family was a commonly mentioned foundation of wellbeing. While highly valued, the ability to buy quality and nutritious food was described as a challenge for many people. This issue was heightened for larger families and those with close social networks, with whom they may share meals.

*Money. It’s expensive to eat right. (Adults, Port Augusta)*

*If you’ve got a big family, it takes a lot more to feed them. (Adults, Tamworth)*

*We have a meat shop here too as well, that sells all kangaroo meat, and the food is so dear. Like, meat in [supermarket] is shocking, so a lot of our people have to go and just buy the cheapest meat …, a lot of people can’t afford it, it’s just probably one every pay week or something. (Adults, Port Augusta)*

*The brain needs protein. Steak. Sometimes it’s expensive. (Youth, Karratha)*

The ability to hunt, grow and collect food directly from the environment on Country was predominantly described by participants living in remote and regional locations as a helpful strategy for ensuring food security in times of food scarcity, while the barriers to these activities were also keenly felt.

*Kangaroo meat where people go around sharing it, they go out and get a hunt and then they bring it back and share around, yes. Because it’s expenses of the food, even fruit and vegetable is so dear. (Adults, Port Augusta)*

Hunting, foraging, and growing food were identified as important in combating food scarcity and when the cost of food is prohibitive. These activities were described as critical in enabling families and communities to source and share known and nutritious foods.

*Go and get kangaroo and cook it out in the sand hills, yes, so you can do that around here. Yes, they go out and get kangaroo meat and bring it back and share it to family, share it to community, the needy. So, someone might want a piece of meat, so that bring that back and share it out. (Adults, Port Augusta)*

Some participants communicated their experiences of food insecurity around the loss of knowledge around traditional foods and/or cooking in traditional ways, along with difficulties associated with access.

*Even our cultural food, our traditional food, not many of us know our traditional ways. Especially with food, what we used to eat. (Adults, Brisbane)*

In discussing their wellbeing, some youth participants expressed gratitude to be living in a household with enough food.

*I feel lucky that I have a fully stocked cupboard because there’s a lot of people out in the world that don’t have that, it’s like a privilege to have food like that. (Youth, Townsville)*

## Discussion

This study conducted a secondary analysis of two large national qualitative datasets about wellbeing, which together comprise the stories of 531 First Nations people (aged 12–92), to explore the ways in which foodways interact with and support wellbeing. Our analysis of the views of the First Nations adults and youth in these datasets revealed that despite the effects of colonisation on First Nations communities and cultures there are deep and inextricable connections between foodways (both traditional and contemporary) and wellbeing for First Nations peoples that are supported via five distinct but interrelated pathways: (1)* connecting with others through food; (2) accessing traditional foods; (3) experiencing joy in making and sharing food; (4) sharing information about food and nutrition; and (5) strategies for improving food security*.

Our central finding, of strong and complex connections between foodways and wellbeing for First Nations Australians, aligns with the few reported findings from similar research conducted both in Australia and with other First Nations populations internationally. Within the Australian context, a qualitative systematic review conducted by Christidis and colleagues synthesizes the literature regarding First Nations peoples’ concerns and priorities regarding food and nutrition in Australia [12]. Christidis and colleagues identified that many First Nations peoples viewed food and traditional foodways as supporting and maintaining health and wellbeing via strengthening connection to culture and identity, as well as via conferring nutritional benefits [12]. The complex transition from traditional to contemporary foodways has been acknowledged in previous studies as posing potentially harmful effects on wellbeing, through the challenges experienced by First Nations people in understanding contemporary foods and the removal of nutritional benefits from traditional foodways, [15] and through loss of autonomy over the food supply [47]. These impacts on wellbeing are also reflected in the results of the current study. However, whilst it is suggested that traditional foodways are linked to cultural and social strength and wellbeing for First Nations peoples in our findings, it was also clear that new foodways can also impact on the wellbeing of First Nations peoples through opportunities for social connection with family and community.

Our finding of *connecting with others through food* is an important pathway through which wellbeing is supported by food and foodways was also noted in Christidis and colleagues’ systematic review [12]. Food has been recognised as playing an important role in the maintenance and experience of holistic health in the wellbeing of First Nations peoples, strongly supported by the connective threads of culture, community, Country and family [6, 11]. The opportunities that foodways provide for social connection among First Nations peoples has been reported in other studies, including through the consumption and harvesting of food, as well as via the places, stories and ceremonies that are associated with food [11, 48]. Furthermore, food has been described as being an important facilitator of cultural engagement [49] and a means by which cultural transmission can be enacted between individuals, groups, and passed down through generations [50].

Traditional diets for First Nations peoples were known to be varied depending on location, nutritionally dense, low in sugar and saturated fat, with procurement of food a very energy intensive process [14]. The changing food landscape for First Nations peoples, as a result of colonial pressures, has decreased the ability to sustain the nutritional and physical aspects of traditional foodways [14]. Our thematic finding of *accessing traditional food* is important to consider in a contemporary context, with practical access to traditional food sources more likely for those in remote areas [14]. Despite the better access to traditional foods in less urbanised settings, there are also continuing colonial, structural and systemic barriers in these settings that prevent access to these foods. Our findings report that First Nations peoples feel that accessing traditional foods on traditional Country offers the greatest benefits to health and wellbeing. This finding aligns with existing evidence of enhanced wellbeing outcomes when First Nations people are able to engage in cultural practices on their own Country [11, 51]. There are, however, a number of barriers that may prevent First Nations peoples from accessing traditional foodways on their own Country (or in some cases aligned Country for which one may have relational connectivity, obligation or relationship). Urbanisation and development, even in rural areas, is a key barrier which reduces access to and function of lands on which traditional foodways have been enacted [52, 53]. The location where First Nations peoples resides also influences this access, with only 27% of First Nations adults reporting residence on their homelands or traditional Country in 2018–19 [54]. Furthermore, huge tracts of land have been made inaccessible for engaging in traditional foodways due to zoning as national parks or wilderness areas [53, 55]. Our findings also echo international research conducted with other First Nations populations, particularly among peoples who have experienced colonisation, where traditional foods are being replaced by less nutritious contemporary foods, food has been harnessed as a colonial tool, and food insecurity is widespread [16, 56–59]. In response to such barriers and concerns, consideration of the extensive history of First Nations peoples’ sophisticated land management should be undertaken to understand how traditional food supplies would and can be preserved with continuation of traditional foodways [52].

With the above barriers to accessing traditional foodways on Country, it is important to reflect on how food continues to act as a conduit for community and social connection in a contemporary context, with participants *experiencing joy in making and sharing food*. Our findings reveal that despite the many challenges to engaging in traditional foodways, First Nations peoples still acquire great wellbeing benefits from engaging in meal and food-related activities with family and friends that are not necessarily a part of traditional First Nations culture. A key example of this is the contributions of youth participants who find social and cultural connection in foodways like pancakes and chocolate covered strawberries with loved ones. With many First Nations peoples now living off Country in urban contexts with reduced access to traditional lands, our finding highlights that there are important opportunities for policy and program design to draw upon First Nations foodways – both traditional and contemporary – as important contributors to wellbeing.

*Sharing information about food and nutrition* emerged thematically in the current study. Our participants spoke to the complexity of contemporary nutrition information, and difficulties around making an informed nutrition choice in a contemporary food landscape. Given the extent of change within foodways for First Nations people in Australia since colonisation, and the resulting loss of some traditional foodways, it is unsurprising this difficulty in navigating food systems has been referenced in previous studies on food choices [15]. Nutrition education programs specific for First Nations peoples have had varying success, with community involvement in the design of the education identified as the most important factor in successful outcomes [60]. Despite the challenges of colonial food-based influences and resulting contemporary foodways, participants in our study referenced family role-modelling and school-based programs as mitigating factors which encouraged healthful food consumption. This mirrors previous studies in which parents encourage positive food behaviours [12, 15, 47]. It has been suggested that centring nutrition knowledge around traditional foodways would assist in First Nations’ peoples having a “meaningful reference point” for education around contemporary foods and help with ameliorating “knowledge out of balance” [15]. An amalgamation of traditional knowledges with contemporary foodways and social connection may be a solution that is reflective of the fabric of Indigenous wellbeing previously described, [6, 9] where culture, community and holistic health are interwoven. Future nutrition education interventions may benefit from such an approach, whilst also acknowledging the essentiality of community consultation and co-design.

The depictions of *strategies for improving food security* within the current study were significant, with food security generally seen as a key contributor to the experience of wellbeing. Traditional foodways and the sharing of food amongst family and community were seen as mitigating factors of the experience of food insecurity, which aligns with previous findings around traditional foods [21]. Further, existing research has identified that the distribution and sharing of food is a common cultural practice among First Nations Australians’ that can provide critical buffering through periods of food insecurity [21, 61, 62]. Similar findings regarding food sharing to improve food security have also been described as supporting the wellbeing of First Nations Canadians [63]. These findings are particularly salient given the high incidence of food insecurity across Australia, especially for First Nations peoples [64]. Current estimates of food insecurity stand at 22% for all First Nations peoples, and 31% for those living in remote communities [64]. The data which informs these statistics is ten years old and based on a brief 2-item tool [65]. Additionally, our adult data was collected in 2017–18, prior to the COVID-19 pandemic, which introduced substantial barriers to food acquirement [25, 66]. The prevalence of food insecurity amongst the general Australian population and First Nations peoples is thus likely to be significantly higher than current estimates, [67] and the impact of food insecurity on wellbeing likely more significant than our current findings. Food security issues are grounded in socio-economic, environmental, systemic and cultural factors beyond the scope of this paper [21]. However, the findings of the current study suggest that there would be value in more broadly considering how access to traditional foods could contribute to ameliorating the growing issue of food insecurity in Australia, as there are likely to be cumulative wellbeing benefits for First Nations people if both food insecurity and traditional food access are considered and addressed together.

Our findings highlight five key pathways through which foodways, both traditional and contemporary, continue to play important roles in determining and supporting the wellbeing of First Nations peoples. These findings provide constructive, nationally relevant evidence to guide and inform health and nutrition programs and services to harness the strengths and preferences of First Nations peoples to support the health and wellbeing of First Nations peoples more effectively. There are current and significant opportunities for our findings to inform the development of improved health and nutrition policy and practice, particularly with regards to food security and equity. On a national level, the Australian Government is developing a strategy for food security in remote First Nations communities, and will shortly be conducting a consultation process [68]. Similar endeavours are currently underway in a number of Australian states, including the development and implementation of a remote food security strategy in First Nations communities in Queensland, [69] a multi sector collaboration in New South Wales working to improve food equity for First Nations peoples, [70] and the development of a regulatory framework to improve food security through stores in remote First Nations communities in the Northern Territory [71]. Understanding and leveraging our findings relating to the pathways between foodways and wellbeing, in partnership with First Nations communities, offers a unique, strengths-based approach to engaging and supporting First Nations peoples’ foodways as a means of promoting health, wellbeing and food equity.

### Limitations and strengths

The strength of the current work lies in the use of decolonising, culturally appropriate qualitative methodologies that privilege the voices of First Nations participants. Both the WM2Adults and WM2Youth studies used a collaborative research strategy with Aboriginal and Torres Strait Islander, and non-Indigenous, researcher contributions. The guidance of Aboriginal and Torres Strait Islander Advisory Groups throughout both projects further ensured privileging of First Nations’ perspectives and voices.

Our large sample of First Nations’ adults and youths from across Australia led to the collection of diverse perspectives around the contribution of foodways to wellbeing. A limitation of our sample was the majority of participants were female and aged 12 -19 years, thus the views from participants in older and male participants may be underrepresented.

## Conclusions

This study has identified a set of pathways through which food and foodways support and promote wellbeing for First Nations Australians. These findings are grounded in the views and perspectives of First Nations peoples and provide evidence of the connections between First Nations foodways and wellbeing. These findings can be used to guide strengths-based collaborative approaches to developing policies and programs. While grounded in the Australian context, these findings highlight the importance of traditional foods and food traditions in supporting the wellbeing of peoples who have endured significant assaults on their culture and identity. This underscores the value in considering the undoubtedly diverse, yet likely influential, pathways by which foodways support wellbeing for many colonised and displaced peoples.

## Data Availability

Data may be available from authors on request. Please contact corresponding author, kate.anderson@uq.edu.au if interested.

## Abbreviations

WM2Adults: What Matters 2Adults
WM2Youth: What Matters 2Youth
